# Abdominal Parietal Metastasis from Cervical Cancer: A Review of One of the Most Uncommon Sites of Recurrence Including a Report of a New Case

**DOI:** 10.3390/life14060667

**Published:** 2024-05-23

**Authors:** Irinel-Gabriel Dicu-Andreescu, Marian-Augustin Marincaș, Anca-Angela Simionescu, Ioana Dicu-Andreescu, Virgiliu-Mihail Prunoiu, Sânziana-Octavia Ionescu, Ștefania-Ariana Neicu, Gabriela-Mădălina Radu, Eugen Brătucu, Laurențiu Simion

**Affiliations:** 1 Clinical Department No 10, General Surgery, University of Medicine and Pharmacy “Carol Davila”, 050474 Bucharest, Romania; irinel-gabriel.andreescu@rez.umfcd.ro (I.-G.D.-A.);; 2 Department of Oncological Surgery, Oncological Institute “Prof. Dr. Alexandru Trestioreanu”, 022328 Bucharest, Romania; 3 Department of Obstetrics and Gynecology, Filantropia Clinical Hospital, 011171 Bucharest, Romania; 4 Department of Pathological Anatomy, Oncological Institute “Prof. Dr. Alexandru Trestioreanu”, 022328 Bucharest, Romania

**Keywords:** parietal, metastasis, cervical cancer, HPV, hysterectomy, radiotherapy, lymph node

## Abstract

Introduction: Cervical cancer is the fourth most common cancer in women, the highest mortality being found in low- and middle-income countries. Abdominal parietal metastases in cervical cancer are a very rare entity, with an incidence of 0.1–1.3%, and represent an unfavorable prognostic factor with the survival rate falling to 17%. Here, we present a review of cases of abdominal parietal metastasis in recent decades, including a new case of a 4.5 cm abdominal parietal metastasis at the site of the scar of the former drain tube 28 months after diagnosis of stage IIB cervical cancer (adenosquamous carcinoma), treated by external radiotherapy with concurrent chemotherapy and intracavitary brachytherapy and subsequent surgery (type B radical hysterectomy). The tumor was resected within oncological limits with the histopathological result of adenosquamous carcinoma. The case study highlights the importance of early detection and appropriate treatment of metastases in patients with cervical cancer. The discussion explores the potential pathways for parietal metastasis and the impact of incomplete surgical procedures on the development of metastases. The conclusion emphasizes the poor prognosis associated with this type of metastasis in cervical cancer patients and the potential benefits of surgical resection associated with systemic therapy in improving survival rates.

## 1. Introduction

Cervical cancer is the fourth most frequent cancer in women worldwide, with about 604,000 new cases and 342,000 deaths reported in 2020 [1]. Almost 90% of cases take place in low- and middle-income countries [2]. The most prevalent cause is chronic infection with human papillomavirus (HPV) [3], favored by a series of additional risk factors such as a weaker immune system caused by coinfection with HIV/AIDS [4,5], obesity [6], smoking [7], multiple sexual partners, multiparity [8], and a diet poor in fruits and vegetables [9]. Also, some studies have shown that women with a first-degree relative who has had cervical cancer may have a higher risk of developing the disease themselves [10].

The treatment options for this type of cancer vary depending on its stage, established by the International Federation of Gynecology and Obstetrics (FIGO). In the early stages (IA1, IA2), the patient can opt for fertility preservation methods such as cervical conization or radical trachelectomy, or, if fertility preservation is not desired, an extrafascial hysterectomy can be performed (also known as hysterectomy type A according to the Querleu–Morrow classification) for stage IA1 or a modified radical hysterectomy (hysterectomy type B) for stage IA2. For stages IB1, IB2, and IIA1, in which the tumor does not exceed 4 cm in diameter and is limited to the cervix, the recommended intervention is a radical hysterectomy type C with pelvic lymphadenectomy [11].

For stages IB3 and IIA2, external radiotherapy combined with concurrent platinum-based chemotherapy followed by intracavitary brachytherapy is recommended. Another alternative is external pelvic radiotherapy associated with concurrent chemotherapy and brachytherapy followed by a complementary radical hysterectomy (type C), but only for certain selected cases. Starting with stage IIB, the NCCN recommends concurrent chemoradiotherapy with the possibility of additional external beam irradiation with 5–10 Gy in case of parametrial invasion, as well as irradiation of the para-aortic lymph nodes [11].

The local guideline of the Bucharest Oncological Institute follows the ESGO/ESTRO/ESP [12] and NCCN treatment guidelines but also provides an alternative treatment option for selected cases of patients with stages considered locoregionally advanced (Stages IIB, III, and IVA) but nonmetastatic. In these cases, our guideline recommends that the concurrent radio-chemotherapy be followed, after an interval of 6–8 weeks, by open radical surgery [13].

The abdominal wall is a complex structure consisting of connective tissue, muscle, fat, and skin. Parietal metastases from internal malignancies range in frequency from 1–4.6% [14], a number that is even lower when it comes to cervical cancer—0.1 to 1.3% [15].

Parietal metastases can involve the skin and can invade conjunctive tissue, and muscles typically appear near metastatic lymph nodes, surgical scars, such as laparoscopic port sites [16], or the umbilicus [17]. They are often a late indicator of the disease, although occasionally they might be the first indication of internal cancers like lung, renal, and ovarian cancers [17]. The most common initial tumors that present parietal metastasis are the breast and ovaries in women and the lung and colon in men [18].

Parietal metastases are divided into two main categories based on the location of the lesion and the past surgical history of the patients: Sister (Mary) Joseph Nodules (SJNs), which are metastatic umbilical tumors [19], and non-SJN skin metastases. SJNs usually develop in patients with gastrointestinal and gynecological cancers [20]. However, umbilical metastases that appear as a port-site recurrence following laparoscopic surgery are not included in the SJNs group. Also, in individuals with peritoneal dissemination, an SJN may appear as the initial presentation of a tumor or as a marker of recurrence [21]. Although chemotherapy can resolve peritoneal dissemination, an SJN may still arise even in the absence of other concomitant peritoneal recurrences [22]. Furthermore, patients with severe involvement of the superficial lymph nodes, such as the axillary and inguinal nodes, may develop SJNs [23].

Based on previous medical treatments or situations, non-SJN parietal metastases can be further classified into three major patterns such as parietal metastasis following surgery, injury, or the presence of lymphadenopathy. After surgery, parietal metastases frequently appear at the location of the incisions following surgery for gynecological and gastrointestinal malignancies and it is claimed that about 1–2% of patients who have undergone laparoscopic surgery for malignant illness have port-site recurrences [24].

Non-SJN metastasis following injury can appear even when the location of a traumatic injury is far from the source of the primary tumor. The literature describes a few such cases: skin metastases occurred at the injection site in a patient with advanced prostate cancer receiving subcutaneous goserelin treatment [25]; also, a patient with colon cancer developed skin metastases at the site of the inflammatory reaction to skin test antigen (Dinitrochlorobenzene) [26]. Last, but not least, a patient with laryngeal cancer who did not have lymph node metastases experienced the development of many superficial nodules encircling the region that previously had the body spica cast applied on from an earlier incident [27].

Finally, skin metastases may appear in the region of the metastatic superficial lymphadenopathy. In some cases, patients with breast cancer experienced skin metastases in the chest wall after axillary node metastases [28]. In patients with prostate cancer, inguinal node metastasis was followed by skin metastases in the lower abdomen, scrotum, and penis [29]. Moreover, in patients with cervical cancer, skin metastases to the vulva, upper thigh, and lower abdomen appeared after inguinal node metastasis [30,31].

**Figure 1:**
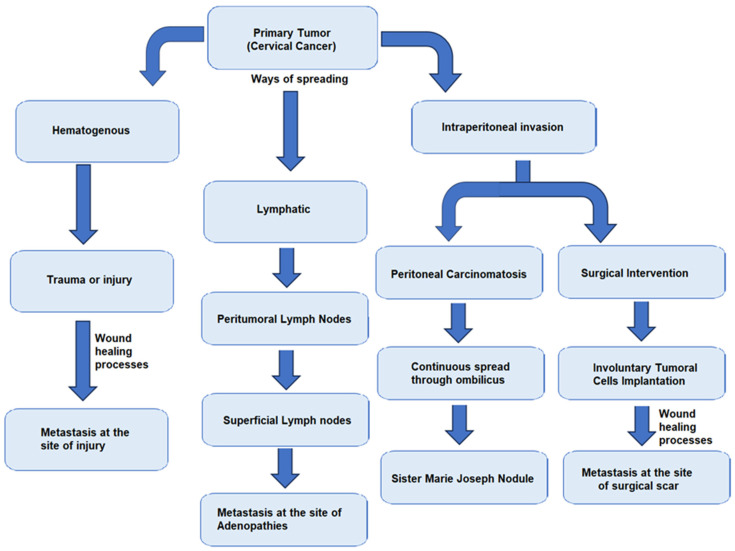
Schematic description of tumor dissemination pathways.

During surgical intervention, certain steps must be taken to prevent the dissemination of tumor cells, such as clamping fallopian tubes at the start of the surgery, washing the abdominal cavity with sodium chloride 0.9% at the end of the operation, and obtaining all lymph nodes and lymphadenectomy specimens without lymph node fragmentation [32]. In Figure 1, there is a brief description of the main pathways of dissemination of tumor cells.

Here, we also present a rare case of abdominal parietal metastasis that occurred 28 months after treatment for stage IIB cervical carcinoma and a review of other similar cases. Informed consent for the research and publication of the data was obtained from the patient according to the Declaration of Helsinki, revised in 2000 in Edinburgh.

## 2. Case Description

A 47-year-old patient was admitted to the Department of Oncological Surgery of the Bucharest Oncological Institute in November 2019 for vaginal bleeding and leucorrhea and was diagnosed with stage IIB adenosquamous carcinoma. From her medical history, we found that she was nulliparous and had frequent episodes of depression that have intensified in the last 3 years, for which she did not take medication.

A multidisciplinary committee consisting of a surgeon, an oncologist, and a radiotherapist was convened, who decided to perform radiotherapy with concurrent chemotherapy as the first step of treatment. Six weeks after the neoadjuvant treatment was completed, only if the patient had a good response, meaning that the tumor had significantly decreased in size or had completely disappeared, radical hysterectomy type C with pelvic lymphadenectomy was recommended.

The patient was directed to the radiotherapy department to perform neoadjuvant radiotherapy with concurrent chemotherapy. The preirradiation CT examinations revealed: an enlarged uterus of 11/7.5 cm, a tumorous cervix of 7/3.5 cm with fine irregular external contour especially on the left lateral circumference, and apparent left parametrial invasion. Some small infracentimetric inguinal and external iliac adenopathies were also described. Twenty-five sessions of external radiotherapy with a total dose of 50 Gray (Gy) and five sessions of concurrent chemotherapy (Cisplatin) (until February 2020) were performed, followed by three sessions of endocavitary brachytherapy (until March 2020).

At the imaging examinations after the completion of radiotherapy treatment (April 2020), the patient showed a favorable response with a decrease in the size of the uterus and the complete disappearance of the cervical tumor, leaving an area of 9 mm of fibrous tissue at the site of the former tumor. Small bilateral external iliac and inguinal lymph nodes were still present. However, the PET-CT examination did not reveal any metabolically active lesions with an oncological substrate.

**Figure 2:**
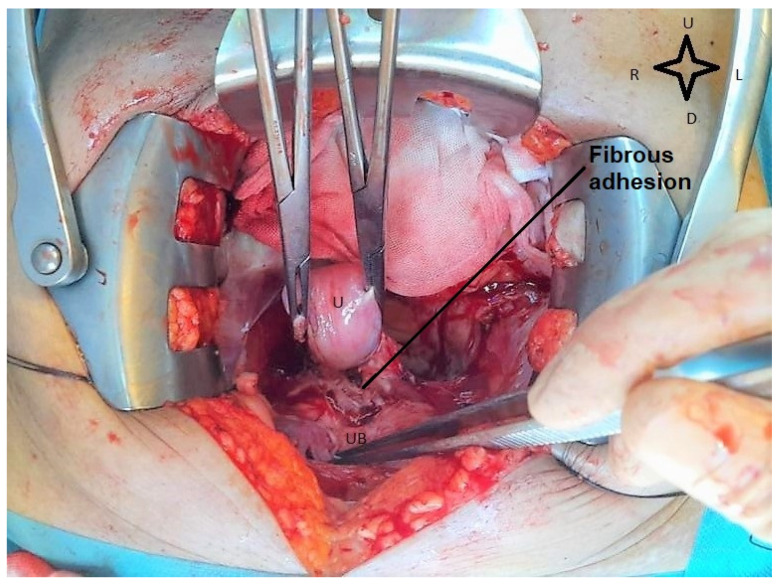
**Intraoperative view during radical hysterectomy.** The cervix is adherent to the urinary bladder. U—uterus; UB—urinary bladder (image from video archive of Bucharest Oncological Institute, personal collection of Dr. Marincaș).

Although the internal guide recommends that the operation should be performed 6 weeks after the end of the neoadjuvant treatment, it was performed after 12 weeks because of the patient’s hesitation, in August 2020. Intraoperatively, a uterus of quasinormal size was visualized, with the bladder intimately adherent to the cervix, (as shown in Figure 2), with slight retraction of the parameter and the left paracolpium. At the level of both ilio-obturator fossae, a fibroinflammatory remodeling process was observed, probably postradiation, without palpable adenopathies.

A radical hysterectomy type B2 according to the Querleu–Morrow Classification (classical “modified” radical hysterectomy) was performed, consisting of excision of the uterus and both adnexa, with the lateral mobilization of the ureter and resection of the nodes of the lateral part of the paracervix with a vaginal cuff of 10 mm. The resection margin was free of tumor cells. Radical lymphadenectomy could not be performed due to fibrotic changes developed postirradiation, but we managed to take biopsies from both ilio-obturator lymph node tissue. To prevent tumor dissemination, both fallopian tubes were clamped at the beginning of the operation, the pelvic cavity was washed at the end, and the sampling of lymph nodes was performed without lymph node fragmentation. At the histopathological examination of the resection piece, no residual tumor was identified at the level of the cervix.

Postoperative, the clinical evolution was favorable and the patient was discharged after five days. No immediate complications were noted. A periodic check-up, including clinical examinations, vaginal swab cytology, and CT and MRI examinations was performed.

Until December 2022, no signs of locoregional tumor recurrence, distant metastasis, or abdominopelvic tumoral adenopathy were detected. However, in March 2023, 28 months after surgery, the patient presented in the clinic complaining of slight pain in the right iliac fossa and the appearance of a 4 cm palpable tumor in the same place, with skin retraction and erythema, a hard consistency, being fixed to the muscle plane, and also painful when the right abdominal muscle was contracted.

**Figure 3:**
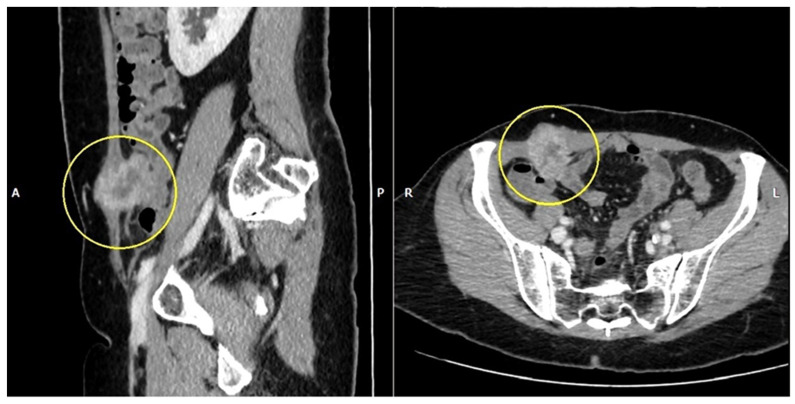
Abdomino-pelvin CT scan shows the parietal tumor in the right iliac fossa (yellow circles).

**Figure 4:**
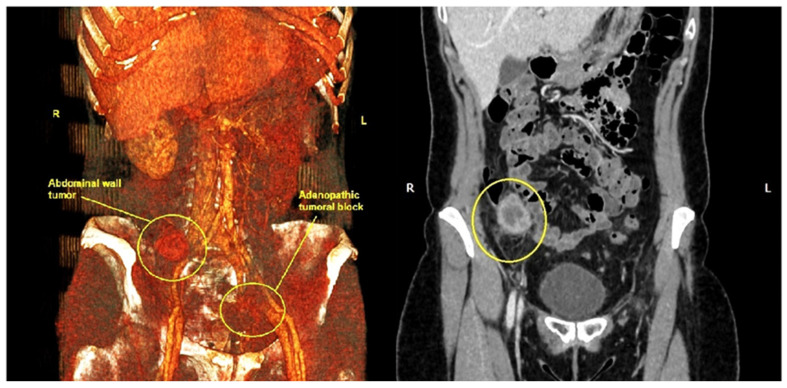
Abdominal CT scan with 3D reconstruction shows the parietal tumor from the right iliac fossa and the left iliac adenopathic block (yellow circles). (R—right, L—Left).

An abdominopelvic CT scan revealed a 37/28 mm tumor in the right iliac fossa in the full thickness of the abdominal wall (Figure 3). Also, there were numerous pelvic lymph nodes in the left ilio-obturator fossa with a tendency to form adenopathic blocks (Figure 4).

**Figure 5:**
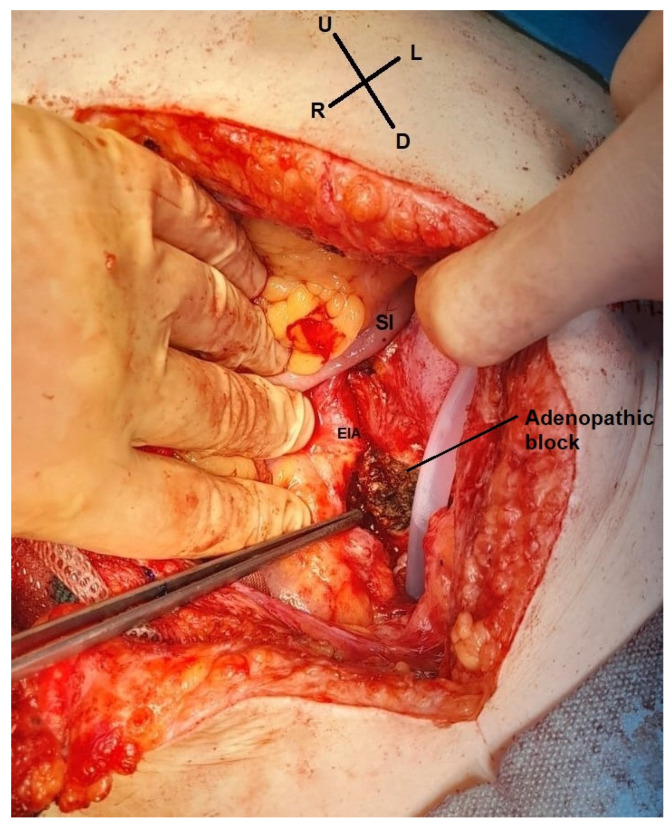
Intraoperative view. The left adenopathic block surrounds the external iliac artery (EIA). SI—small intestine (image from video archive of Bucharest Oncological Institute, personal collection of Dr. Marincaș).

The multidisciplinary committee decided that surgery should be the next step of treatment due to the benefits it can bring on the histological result and also through the removal of the painful right iliac fossa tumor. Intraoperative findings included a 4.5 cm parietal tumor, corresponding to the scar of the former abdominal drain tube at the level of the right iliac fossa, with the great omentum adhering to it, without any other intraperitoneal metastases. We also found a left iliac adenopathic tumor block surrounding the left external iliac vein (Figure 5), which was biopsied, as it could not be completely resected due to the vascular risks involved. A pelvic drain tube was placed at this level as shown in Figure 5.

**Figure 6:**
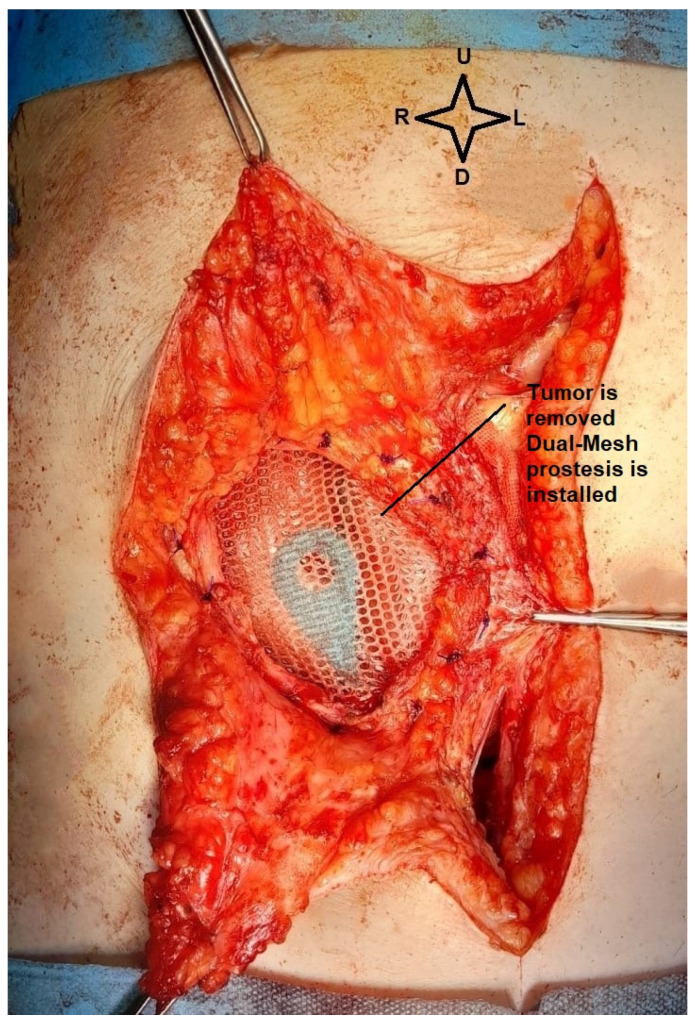
Intraoperative view. The dual mesh prosthesis, with the small intestine below (image from video archive of Bucharest Oncological Institute, personal collection of Dr. Marincaș).

Unfortunately, relevant pre-excisional images of the parietal tumor could not be taken as it crossed the entire abdominal wall with a small area of skin retraction and intense adhesions to the omentum. Excision of the parietal tumor was performed with 1 cm safety margins around and the abdominal wall was reconstructed using a textile Dual Mesh prosthesis. Below in Figure 6, we present the parietal defect left after tumor excision.

**Figure 7:**
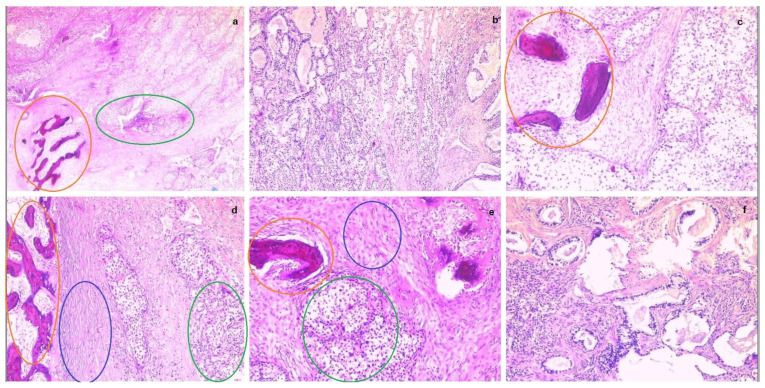
From (**a**–**f**) parietal tumor fragments in different magnifications, showing malignant infiltration (adenosquamous carcinoma) with areas of ossification. (Ossification areas—orange circles, glandular tumor cells—green circles, squamous tumor cells—blue circles).

The intraoperative histopathological result of the parietal tumor was an adenosquamous carcinoma with areas of ossification. The left iliac adenopathic block shared the same histopathological result. Figure 7 below shows various sections of the parietal tumor in different magnifications.

The postoperative recovery was favorable again with the drainage tube suppressed on the third day after surgical intervention. The patient was discharged home six days later in good general condition, with the wound healing. However, when she returned at 30 days for re-evaluation taking into account the final histological result of adenosquamous carcinoma metastasis, the multidisciplinary committee recommended the continuation of the chemotherapy treatment, an option that the patient firmly refused.

After another 36 days, she presented to the emergency room asthenic, underweight, and complaining of moderate joint and bone pain. The next day, unfortunately, the patient requested to be discharged for personal reasons before any further evaluation. In the following week, we tried several times to contact her by phone but without success. After ten days from discharge, we found out that she died at home.

## 3. Discussion

Parietal abdominal metastases in cervical cancer are very rare with less than 20 cases reported in recent decades. Implementing HPV vaccines for females and males, early detection of cervical dysplasia including the Pap smear, liquid-based cytology, and high-risk HPV identification test has been shown to reduce the incidence and mortality of cervical cancer in the United States of America [33,34].

A way to avoid the reluctance of patients to screening through classical methods (cervical cytology) can be the wide spread of self-testing devices for HPV detection [35]. Also, the education of patients regarding the risks and benefits of screening methods is deficient. Moreover, genital diseases are often seen as shameful topics for discussion so the stages of presentation are usually advanced, and associated with symptoms such as vaginal bleeding, vaginal pain, or abnormal vaginal discharge.

Table 1 presents a summary report of some of the most representative cases of abdominal parietal metastasis. We can observe that the most frequent places of occurrence of metastases are the pelvis, chest, and lower limbs. Also, the stages usually associated with skin metastases are IIB and above, and, in most cases, they involve multiple areas of the body. 

In addition, as expected, the more advanced the stage of the disease, the shorter the period of occurrence of metastases, with a period of 1–2 months in stage IIIB. The prognosis is very poor, with a survival period of 1 and 7 months at most after the appearance of metastases, with an overall 5-year survival of 17% [41].

We also discussed the case of a 47-year-old patient who initially presented with vaginal bleeding and leucorrhea. These are usually the most common symptoms when it comes to genital neoplasia [42]. The result of the patient’s cervical biopsy was cervical adenosquamous carcinoma. This histological type is quite rare, with an incidence of only 5–10% of the total number of cases, and it is also associated with a poorer response to neoadjuvant treatment [43]. According to some studies, the response of this histological type is only 55–60%, so after neoadjuvant treatment, 45% of patients will still have residual tumor cells at the level of the cervix [44].

This case, in particular, emphasizes the importance of pelvic lymphadenectomy in assessing the lymph node status in patients with cervical cancer. Lymph nodes can act as a potential reservoir of tumor cells even after neoadjuvant treatment. It also shows the importance of the time recommended by the local guidelines (six weeks after the nonadjuvant treatment) in order to avoid post-irradiation fibrosis, which may limit the optimal extent of surgical intervention.

The imaging examinations before neoadjuvant treatment showed a cervical tumor with a diameter greater than 4 cm with an apparent invasion of the left parameter without reaching the pelvic wall, which is consistent with stage IIB. The reported 5-year survival rate at this stage is 63.9% [45].

The Guide of the Oncological Institute of Bucharest follows the ESGO/ESTRO/ESP [12] and NCCN treatment guidelines but also maintains a treatment alternative for locoregionally advanced (IIB, III, IVA) but nonmetastatic cervical cancers. This is represented by the performing of open surgical intervention six weeks after the completion of neoadjuvant treatment (radio-chemotherapy), consisting of radical hysterectomy type C whenever possible [13]. In this case, the entire neoadjuvant treatment scheme (radio-chemotherapy and brachytherapy) was carried out according to international guidelines. However, due to the patient’s hesitation and other personal issues, the surgical intervention was performed 12 weeks after neoadjuvant treatment. Due to post-radiation sclero-inflammatory changes, only a type B hysterectomy was performed (the uterus and both ovaries were excised with lateral mobilization of the ureter and resection of the nodes of the lateral part of the paracervix, with a vaginal cuff of 10 mm).

The impossibility of performing the surgical intervention in its entirety due to postirradiation fibrosis is a relatively common adverse reaction; according to some studies, grade 1 and 2 perivaginal fibrosis and stenosis are found in 21.7% of patients with EBRT (external beam radiotherapy) [46]. In this case, the surgery was limited to lymph node sampling with the excision of only three or four lymph nodes on each side, which is considered insufficient to reflect the real state of lymph node invasion. This can represent a negative prognostic factor since the sampling of at least 10 pelvic lymph nodes can help to better stage the disease [47].

As suggested by the imaging investigations after neoadjuvant treatment and later by intraoperative anatomopathological examination, no residual tumor could be identified, which means that the patient had a complete therapeutic response. However, given the fact that the patient presented perivaginal fibrosis that did not allow the resection of the upper third of the vagina and the impossibility of performing pelvic lymphadenectomy, although there was no more tumor tissue, we can consider that the absolute radicality of the intervention was not reached [48].

The patient was in perfect health without any signs of recurrences for almost two and a half years of follow-up but after she presented directly with a parietal mass localized in the right iliac fossa, the imaging investigations also described a left iliac adenopathic block. According to some studies, this is the typical period of tumor recurrence, local or metastatic, which occurs between the second and third year post-treatment [49]. On the other hand, it is a very rare situation for metastasis to appear firstly subcutaneously, in cervical cancer the prevalence being between 0.1–1.3% [15].

Usually, there are two phases in the process of parietal metastasis: the first is the dissemination of the tumor cells to the abdominal wall and the second is the growth of the tumor cells at the new location, which is favored by inflammation, wound healing, and the presence of adipose tissue. Some studies show that tumor cells exploit the healing mechanisms of surgical wounds or other injuries [50]. Mesenchymal, epithelial, endothelial, and immune cells interact through cytokines and growth factors for tissue restoration but tumor cells use these stimuli for their own proliferation [51]. Another factor is the presence of adipose tissue. Some studies claim that around metastases, adipocytes are smaller in size and have a lower lipid content; the tumoral cells use them as a source of energy [52,53]. This fact can explain the rapid progression of metastases for our patient in just 3 months after the periodical check-up, with the abdominal wall having a well represented adipose tissue. In addition, because the tumor was on the site of the old drain tube, it can be argued that the healing mechanism after the drain tube was removed also enabled the recurrence.

Another aspect that has to be discussed is that, in some cases, young patients who want to avoid the unwanted side effects of irradiation on the ovaries and those of early menopause induced by treatment can opt for ovarian transposition intervention, which involves preserving ovarian function by repositioning the ovaries out of the field of radiation. This intervention is generally performed laparoscopically. However, laparoscopic interventions, especially in advanced locoregional stages (IIB and above) have increased risks of tumor cell dissemination [54]. We did not opt for this therapeutic strategy in our patient because she understood all the risks and benefits and accepted radio-chemotherapy as the first treatment.

However, the fact that, after the recurrence of the disease, the patient refused the chemotherapy treatment can be attributed, in part, to the depression that she has had since she was first diagnosed with cervical cancer, with the worsening of symptoms in the last period. According to the latest studies, the prevalence of depression in patients with cervical cancer who received neoadjuvant treatment is 72%, this relation varying according to the quality of life and marital relations [55]. Depressed patients may have problems in compliance with treatment and also it can influence their general clinical condition and quality of life [56].

We believe it is of great importance to provide cancer patients with a variety of resources for multidisciplinary care, such as the aid of psychosocial services during and after the completion of cancer treatment [57]. According to some studies, cancer patients have stated that they generally tend to get oncology-related information or psychological support on their own, rather than from medical staff, after treatment is completed [58]. Therefore, the medical teams should provide more comprehensive post-treatment care through referrals to appropriate psychosocial services.

## 4. Conclusions

Surgery after neoadjuvant treatment for patients with advanced locoregional nonmetastatic cervical cancer (stages IB3–IIB) is practiced in some countries in Eastern Europe with a high incidence of this type of cancer. In our Oncological Surgery Department, an open Type C radical hysterectomy is recommended six weeks after the neoadjuvant treatment if the patient has a complete response to the treatment and good physiological status. However, the fibroinflammatory changes after neoadjuvant treatment can represent an impediment to the radicality of the surgical intervention. Also, a fact of utmost importance, but also limited by the fibrous and inflammatory changes, is the proper evaluation of the lymph node status as they can represent a reservoir for tumor cells and, therefore, the starting point for subsequent dissemination.

Parietal metastases are a very rare entity, especially for gynecological cancers such as cervical cancer. The occurrence of metastasis is also related to the histological type of cancer, some types being more aggressive than others. In the case of our patient, adenosquamous carcinoma, although not so common, is associated with a poorer response to neoadjuvant treatment.

The spreading pathways of parietal metastasis are varied and include dissemination by contiguity, hematogenous, lymphatic, or direct involuntary implantation of tumor cells during surgery. Local factors such as cytokines or growth factors secreted after injuries promote tumor proliferation. Adipose tissue also contributes to tumor growth, being a source of energy. The average time duration of occurrence of metastases is between two and three years after treatment, as was the case of our patient. The overall survival rate is poor, falling to 17% at 5 years, a fact that should encourage further research in order to find the optimal therapeutic strategies.

## Figures and Tables

**Table 1:** Summary of reported cases of cervical cancer with abdominal parietal metastasis.

No	Author	Age	Neoadjuvant Therapy	Surgery	Stage of Disease	Time of Recurrence (Months)	Location of Metastasis	Survival after Metastasis (Months)
1	Agrawal et al. [36]	66	Yes	No	IVA	2	Pelvis	6
2	Basu et al. [37]	60	Yes	Yes	IIA	12	Pelvis, lower thigh	7
3	Benoulaid et al. [38]	63	Yes	No	IIIB	6	Chest, Pelvis	2
4	Malfetano et al. [39]	59	Yes	No	IIIB	1	Pelvis	1
5	Malfetano et al. [39]	58	Yes	Yes	IB	60	Pelvis	1
6	Katiyar et al. [40]	60	Yes	No	IIA	86	Pelvis, back, thigh	1

## Data Availability

The patient’s data were obtained from the medical documents of the Bucharest Oncological Institute and they cannot be made publicly available as they contain personal and confidential data of the patient but any information about these documents can be obtained on request from the corresponding author.
